# Gallstone Ileus: Clinical Presentation and Radiological Diagnosis

**DOI:** 10.7759/cureus.42059

**Published:** 2023-07-18

**Authors:** Shruti Gaikwad, Mandar Marathe

**Affiliations:** 1 Emergency Medicine, Leicester Royal Infirmary, Leicester, GBR

**Keywords:** rigler’s triad, gallstone ileus, gallstone diseases, abdominopelvic ct scan, intestinal obstrution

## Abstract

The term "gallstone ileus" refers to intestinal obstruction brought on by a gallstone lodged within its lumen. The gallstone travels through a fistula that develops because of the constant pressure it exerts on the gall bladder. The symptoms are vague and confounding which can commonly lead to delay in diagnosis. The preferred imaging technique is a computed tomography scan. The diagnosis is confirmed by the identification of Rigler's Triad on a CT scan, which includes a small intestinal obstruction, pneumobilia, and an ectopic stone in the intestine. The condition is associated with several complications and needs to be treated with emergency surgery. This case demonstrates how a patient could have non-specific symptoms and how early detection by imaging was crucial to the patient's treatment.

## Introduction

Gallstone ileus is an uncommon cause of intestinal obstruction, and this study highlights the clinical and radiological findings to build awareness of it. Gallstones develop due to the crystallization of fats and minerals within the gallbladder [1]. Approximately 1-2% of patients experience complications of gallstones and require surgery each year, while more than 80% of carriers are unaware of their gallbladder condition [2]. A mechanical blockage brought on by gallstone impaction inside the gastrointestinal system is known as gallstone ileus, an uncommon consequence of cholelithiasis [3]. It accounts for 1-4% of cases of mechanical intestinal obstruction and is more prevalent in elderly females [3,4]. It is associated with several complications and has a mortality rate of 26.1% [5]. It necessitates caution since it can be easily missed and relies on radiology to make the diagnosis.

## Case presentation

Clinical features

A woman in her 80s who had intermittent, dull, 5/10 severity abdominal pain and episodes of vomiting for one week was seen in the emergency department. She gave a history of being unable to open her bowels for the last four days. There was no prior history of diarrhea or fever. She was known to have chronic lymphoid leukemia (CLL) and hypertension with no previous diagnosis of gallstones. She was not on any treatment for her chronic lymphoid leukemia. On assessment, she was normotensive with a blood pressure of 120/66 mmHg, afebrile, and had a heart rate of 90 beats/minute. On examination, her abdomen was soft, not distended, and the left upper quadrant was mildly tender. No guarding or rigidity was felt. Bowel sounds were heard more frequently. The rest of her examination was unremarkable.

Investigations

The patient already had her blood tests taken upon admission to the emergency department which included the venous blood gas (VBG), full blood count, urea and electrolytes, c-reactive protein (CRP), liver function tests, serum amylase, and acute kidney injury (AKI) score. Her blood revealed that she had developed metabolic alkalosis, raised inflammation markers, and an AKI score of 2 (Table 1).

**Table 1:** Laboratory blood results.

Blood tests	Results	Reference range	Unit
pH	7.579	7.320-7.430	-
Bicarbonate	34.2	20.0-28.0	mmol/L
Lactate	2.4	0.6-1.4	mmol/L
White blood cells	42.4	4.0-11.0	×10^9^/L
Neutrophils	20.78	1.50-7.5	×10^9^/L
C-reactive protein	204	0-10	mg/L
Creatinine	163	60-120	μmol/L

**Figure 1:**
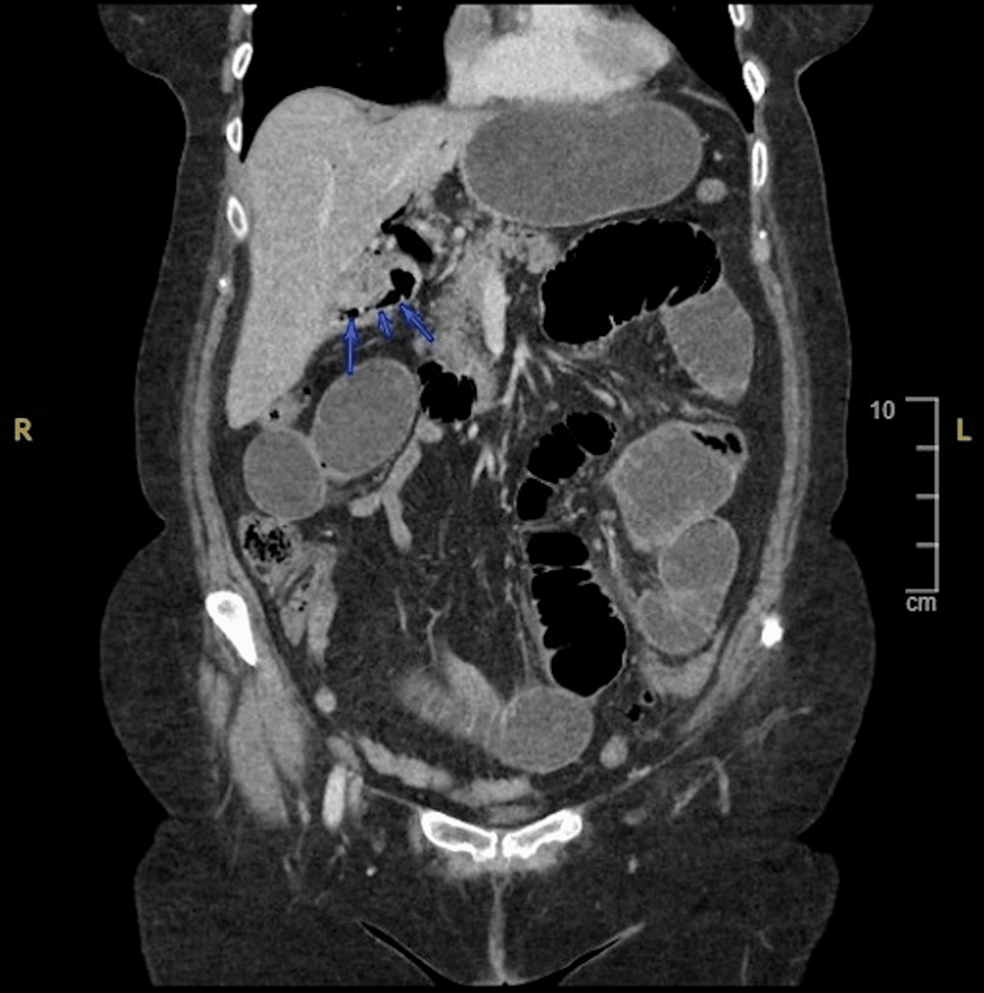
Computed tomography scan coronal view of abdomen showing gas in biliary tree and gallbladder.

**Figure 2:**
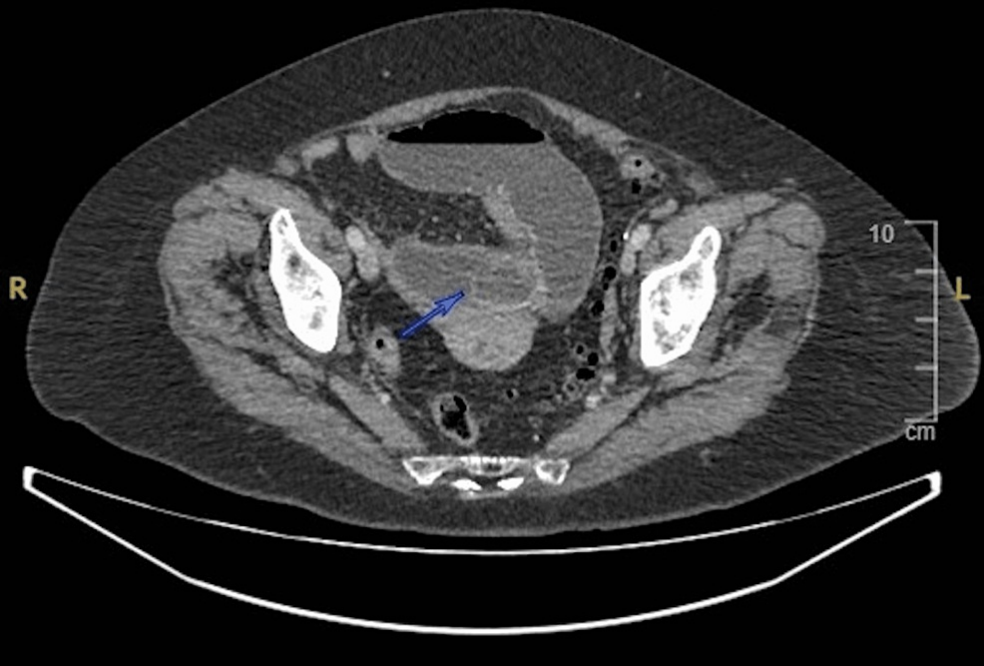
Computed tomography scan axial view of abdomen showing obstructive gallstone in pelvic loop of mid ileum with features of small bowel obstruction.

It is noteworthy that despite the patient's leukemia, these numbers were higher than her baseline. We decided to get a computed tomography (CT) scan of the abdomen and pelvis which revealed gas in the biliary tree and gas locules in the pancreas. A gallstone that was significantly blocking the small intestine was found in the pelvic loop of the ileum and the gallbladder appeared collapsed with air within. Additionally, a fistula between the duodenum and the gallbladder was found. The stomach was distended, and the esophagus was observed to be fluid-filled. Through the CT scan, all three components of Rigler’s triad were identified, which led to the diagnosis of gallstone ileus (Figures 1, 2).

Differential diagnosis

The presenting complaints and examination findings were rather ambiguous, as is evident in the case presented above. In contrast to the abnormal blood test findings, the symptoms were vague and understated. We thus considered a variety of differentials, from simple conditions like viral gastroenteritis to more acute conditions like bowel obstruction or mesenteric ischemia. As she had a week-long history of vomiting, stomach discomfort, and minor abdominal tenderness in addition to elevated neutrophil, we considered gastroenteritis as one of our differential diagnoses. Even though she had reoccurring constipation, her history of being unable to open her bowels for four days prompted us to investigate intestinal/bowel obstruction as a differential. The combination of the aforementioned characteristics and an elevated lactate level aroused suspicions about mesenteric ischemia. Imaging was employed to rule out differentials before making the final diagnosis.

Treatment

As we suspected bowel obstruction, the patient was directed to refrain from any oral intake. She was given Hartmann’s solution intravenously for her acute kidney injury. To decompress and prevent aspiration of gastric contents, a nasogastric tube was inserted, which relieved her symptoms considerably. A referral to surgery was made and the patient was moved from the emergency department to the surgical triage unit. The patient then underwent an emergency laparotomy for gallstone ileus. The procedure was abandoned because the patient became hemodynamically unstable and had to be transferred to the intensive care unit (ITU). There was 1.5 m of small bowel remaining. The patient went back into surgery three days later for anastomosis and drain placement. Postoperatively, the patient was stepped down to the intensive care unit as she developed sepsis. She was started on intravenous meropenem and a peripherally inserted central catheter (PICC) line was inserted for total parenteral nutrition. Four to five days later, the patient became hypoxic, and a computed tomography pulmonary angiogram (CTPA) was done which revealed a bilateral pulmonary embolism. The patient was started on therapeutic dalteparin after advice from a hematologist. A dietician referral was done for nasogastric tube feeding, as the patient was unable to tolerate oral foods initially. The occupational therapist and physiotherapist reviewed the patient as well. The patient required around 42 days to recuperate following surgery before being discharged to a community rehabilitation center.

Outcome

The patient has been discharged from the community rehabilitation center and is recovering at home. An outpatient follow-up appointment with general surgery has been scheduled. Moreover, a follow-up appointment with the general practitioner has been planned to monitor the anticoagulation treatment she is receiving for her pulmonary embolism.

## Discussion

**Figure 3:**
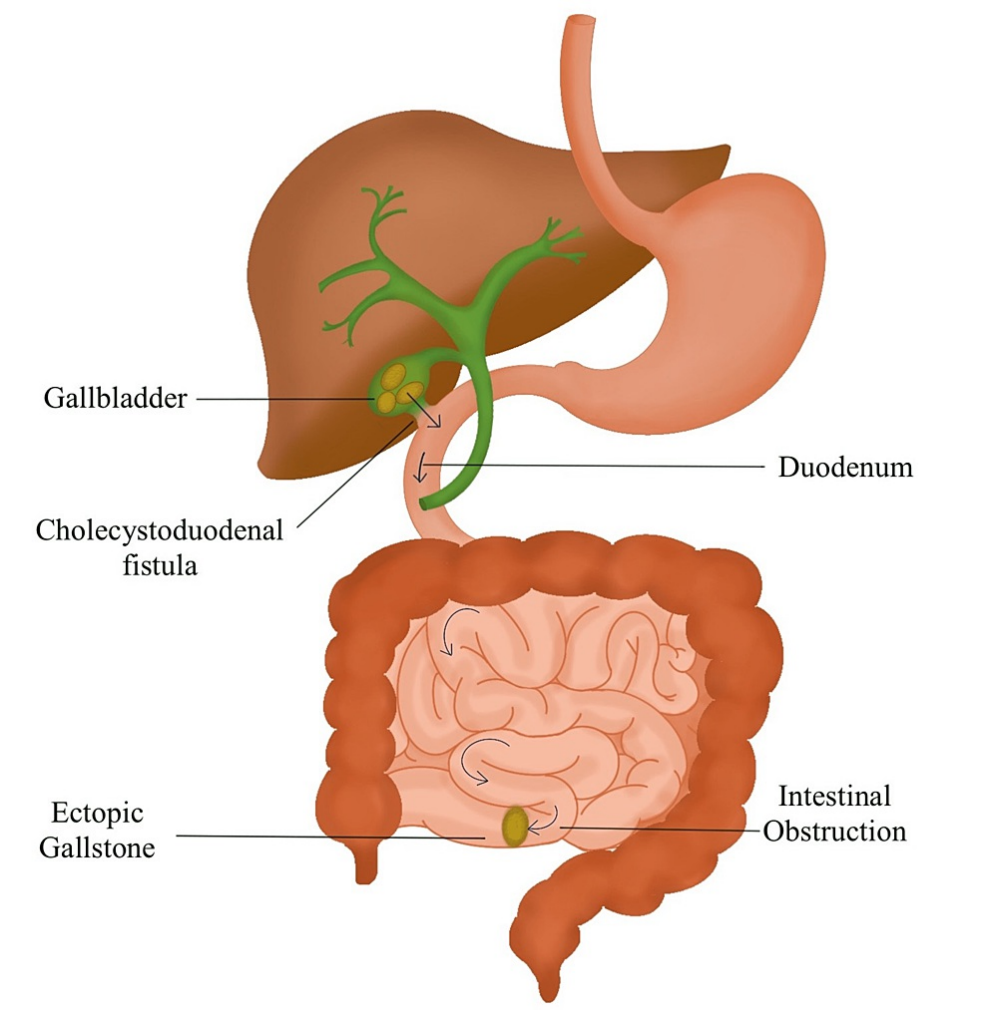
The pathogenesis of gallstone ileus. The illustration depicts the formation of cholecystoduodenal fistula and passage of the gallstone into the small intestine resulting in small bowel obstruction. The illustration is created by the author (Shruti Gaikwad) of this study.

The primary pathological mechanism of gallstone ileus is a biliary-enteric fistula [6]. A fistulous connection between a necrotizing gallbladder and the gastrointestinal tract allows the gallstone to enter the intestine (Figure 3) [3]. Fistula formation results from erosion brought by the gallstone pressing against the biliary wall [7]. The terminal ileum and the ileocecal valve are the most frequent trouble spots for gallstone impaction because of their anatomically small diameters and less efficient peristalsis, according to Reisner and Cohen, who investigated 1,001 instances with gallstone ileus [8]. The jejunum, the Treitz ligament, the stomach, and the duodenum and colon are also uncommon sites of impaction [8]. The clinical manifestations of gallstone ileus are vague and confounding, and it is possible to miss them [9]. Before admission, intestinal blockage symptoms may have been present for one to seven days [10]. The word “tumbling blockage” is often used to describe the halting and rolling descent of the gallstone down the intestinal canal [10]. After the initial vomiting of upper intestinal contents, the vomitus turns feculent, demonstrating the pathology [10].

It is widely recognized that diagnostic radiography plays a crucial role in gallstone ileus. The importance of a careful preoperative diagnosis has been emphasized throughout history, starting with the early experience of a handful of cases detected on abdominal radiography, in which, air in the biliary tree and faintly calcified gallstones were reported, to the first instance discovered in 1983 on CT [11,12]. Along with the Rigler's radiologic trinity, which includes signs of an ectopic stone in the intestine, pneumobilia and/or air within the gallbladder, and small-bowel obstruction, a CT enables direct visualization of the biliary-enteric fistula in addition to pinpointing the precise location of the ectopic stone and the site of obstruction [13]. The dimensions of the impacted gallstone can be estimated using CT as well [14]. Overall, CT had a sensitivity, specificity, and accuracy of 93%, 100%, and 99%, respectively, for identifying gallstone ileus [15]. It must be noted that the triad's three elements may not be identified in all cases [15]. According to research by Yu et al., ectopic stones were visualized on CT scans in 92.8% of cases whereas pneumobilia could only be seen in 50% [15]. Thus, it is essential to interpret CT scans in light of clinical features.

Through early diagnosis and timely treatment, the once-high death rate has fallen to 26.1% during the past few years [5]. Fluid resuscitation and stomach decompression using a nasogastric tube are the first lines of treatment [16]. Additionally, there is a restriction on all oral consumption [5]. Gallstone ileus requires emergency surgery and enterolithotomy is the preferred procedure [17]. Nevertheless, the bowel should be resected with an end-to-end anastomosis if the occluded intestinal portion is not viable [17]. In some cases, laparoscopic-assisted enterolithotomy has been employed as a treatment and has been reported to be more successful [18]. The most common complication of gallstone ileus is acute renal failure, which our patient also developed [19]. Sepsis, urinary tract infection, wound dehiscence, biliary fistula, anastomotic leak, intra-abdominal abscess, and death are other prevalent consequences [20]. The main takeaway is to consider gallstone ileus when making a differential diagnosis, especially in the elderly. Due to our patient’s vague symptoms, history of constipation, and lack of past gallstone history, other differential diagnoses seemed more plausible. While it may present with seemingly innocuous symptoms, if it is not promptly diagnosed, it can be fatal. Thus, it is crucial to have a high clinical suspicion and more awareness of the condition.

## Conclusions

Gallstone ileus is brought on by the halting passage of the gallstone through the intestine and can present with vague symptoms, such as intermittent abdominal pain and vomiting. These symptoms might be present for one to seven days before admission. Other prevalent conditions, such as gastroenteritis, gastritis, and constipation also present with similar symptoms. Thus, those with these complaints may not always go to the emergency department where testing is accessible. Hence, especially in older people, it is crucial to have a high level of suspicion for the more dangerous causes of similar symptoms, such as intestinal blockage. Early referral to the emergency department and prompt diagnosis by imaging can result in quick treatment and the avoidance of complications and even death.
